# Knowledge and Attitude Regarding Gestational Diabetes Mellitus Among Pregnant Women in Tabuk City, Saudi Arabia: An Exploratory Study

**DOI:** 10.7759/cureus.48151

**Published:** 2023-11-02

**Authors:** Manal Hussein Wafa, Afnan I Ayoub, Tayf A Bukhari, Abdulaziz A Amer Bugnah, Abeer Ali H Alabawy, Abdullah H Alsaiari, Hadeel M Aljondi, Safaa H Alhusseini, Ftoon A Alenazi, Hayat M Refai

**Affiliations:** 1 Obstetrics and Gynecology, King Salman Armed Forces Hospital, Tabuk, SAU; 2 College of Medicine, Batterjee Medical College, Jeddah, SAU; 3 Obstetrics and Gynaecology, King Saud Bin Abdulaziz University for Health Sciences, Jeddah, SAU; 4 College of Medicine, Najran University, Najran, SAU; 5 Pediatrics, Faculty of Medicine, University of Tabuk, Tabuk, SAU; 6 College of Medicine, King Abdulaziz University, Jeddah, SAU; 7 College of Medicine, Taibah University, Al-Madinah Al-Munawwarah, SAU; 8 College of Medicine, King Saud University, Riyadh, SAU; 9 Internal Medicine, King Salman Armed Forces Hospital, Tabuk, SAU

**Keywords:** pregnancy, knowledge and attitude, gestational diabetes, tabuk city, pregnant women

## Abstract

Introduction: Gestational diabetes mellitus (GDM) refers to any stage of glucose intolerance that begins or is first noticed during pregnancy. GDM has long been an issue in Saudi Arabia. When a pregnant woman who does not already have diabetes is unable to produce enough insulin, GDM develops. GDM patients not only run the danger of developing a number of health issues for themselves but also for the health of their developing fetus. The first step in GDM screening during pregnancy is raising awareness of the condition.

Methods: This was a cross-sectional study conducted to assess knowledge and attitudes with regard to GDM among pregnant women in Tabuk City, Saudi Arabia. The sample size was 539 females from obstetrics and gynaecology clinics in civil and military hospitals. Data collection was done using a valid questionnaire.

Results: A total of 539 women were included in the study, spanning various age groups from under 20 to above 40 years, with pregnancy occurrences ranging from one to four times. Most participants exhibited strong understanding, with 410 (76.1%) demonstrating awareness of GDM, and 382 (70.9%) having a clear grasp of its definition. Additionally, a majority displayed positive attitudes toward managing GDM.

Conclusion: The Saudi women who participated in this study showed good knowledge of GDM and its risk factors, as well as a good attitude regarding the management of GDM and lifestyle modification to reduce its complications. The participants gave adequately logical answers about the sources of information about GDM and about the barriers to effective GDM management. A highly significant association was noticed between knowledge and attitude regarding GDM among the participants (p = <0.001).

## Introduction

The most common metabolic disorder discovered during pregnancy is gestational diabetes mellitus (GDM), a significant public health concern. GDM has been associated with both short-term and long-term detrimental effects on the health of the mother and the fetus. Some common newborn issues include macrosomia, hypoglycemia, and delivery trauma [1,2]. GDM increases the risk of cardiovascular disease and type 2 diabetes mellitus (T2DM) in the mother [3]. Both modifiable and non-modifiable risk factors are associated with GDM. Age, a history of diabetes in the family, genetics, and race have all been identified as non-modifiable risk factors for GDM [4].

Globally, the incidence of GDM is roughly 7%, while the prevalence may range from 1% to 14% of all pregnancies [5], depending on the population studied and the diagnostic techniques utilized. The prevalence rates differ significantly among countries [6]. For instance, according to recent studies, GDM is more common in Norway and the United Arab Emirates (UAE) than it is in Mexico [7,8]. The incidence of GDM in the Kingdom of Saudi Arabia ranges from 8.9% to 12.5%, according to a study published in 2000 [9].

Pregnancies with GDM are more likely to result in maternal or fetal problems, underscoring the importance of this problem and the necessity of GDM prevention. Pregnant women and their unborn children are more likely to experience risks like a second-trimester miscarriage, preterm birth, preeclampsia, cesarean section, macrosomia to shoulder dystocia, trauma during delivery, stillbirth, neonatal hyperbilirubinemia, hypoglycemia, hypocalcemia, polycythemia, respiratory distress syndrome, and hypertrophic cardiomyopathy [10,11].

It is known that GDM has a stronger correlation with prenatal depression than does a typical pregnancy. Women with GDM are more likely to acquire T2DM, GDM in subsequent pregnancies, cardiovascular morbidity, and death in later life [12,13]. Furthermore, their descendants have a substantially increased chance of developing metabolic syndrome, becoming obese as children, and having impaired glucose metabolism [14].

Health literacy requires knowledge, and knowledge is linked to significant health consequences. It is essential to comprehend GDM to manage it and create preventive actions that will decrease its load. This information should concentrate on its risk factors and effects [15]. It is essential to extensively study the sources of information regarding GDM that expectant women rely on to direct their behavior and attitudes toward disease screening and management. Such details would help them understand the condition better [16].

The prevalence of GDM has been sharply increasing in the Kingdom of Saudi Arabia [15]. GDM screening will therefore present a perfect window of opportunity for the prevention of DM in two generations. Prenatal women's understanding of GDM will make it simpler to adopt a healthy lifestyle, increase healthcare-seeking behavior, and hence enhance sickness prevention and early diagnosis. Our study aimed to evaluate the knowledge of GDM, risk factors, screening, and short- and long-term effects among pregnant women who attended our antenatal clinic.

## Materials and methods

This cross-sectional study was conducted to assess the knowledge and attitudes regarding GDM among pregnant women in Tabuk City, Saudi Arabia. Data were collected from obstetrics and gynecology (OBGYN) clinics in both civil and military hospitals, selected through a random sampling technique, from July 1 to September 30, 2023.

Tabuk City is located in the northwestern region of Saudi Arabia. It has a population of approximately 534,893 people, according to the latest estimates from the General Authority for Statistics in Saudi Arabia. The study population comprised all pregnant women attending OBGYN clinics in both civil and military hospitals in Tabuk City, aged 18 years and older. Pregnant women with pre-existing diabetes, including GDM, were excluded from the study.

Sample size 

The sample size for this study was calculated using the following formula:

$n = \frac{(Z^{2} * P * Q)}{d^{2}}$

where: n = sample size; Z = z-score corresponding to the level of confidence desired (e.g., 1.96 for 95%CI); P = expected prevalence of adequate knowledge and positive attitudes towards GDM management (assumed to be 50%); Q = 1 - P; d = margin of error (assumed to be 5%).

Assuming a 10% non-response rate, the final sample size was 539 pregnant women.

Sampling technique

Participants were randomly selected using a systematic random sampling technique. Specifically, every second individual attending the OBGYN clinics during the study period was included. This approach was adopted to guarantee the representativeness of the sample, ensuring it accurately reflected the population, and to secure an ample number of participants for the study.

Data collection tools

To gather data, we employed a structured interviewing technique utilizing a questionnaire developed through a meticulous review of previous research and expert opinions. The detailed questionnaire can be found in the appendix. Notably, the questionnaire was originally crafted in English. To ensure consistency in data collection, our data collectors underwent training in Arabization techniques during a specialized workshop. This training was specifically designed to eliminate discrepancies in data collection. Prior to the main study, the questionnaire underwent a rigorous pre-testing phase involving 25 participants from the target population who were not included in the actual study. This pilot study was conducted to assess the questionnaire's clarity, relevance, and validity. The results were encouraging, with Cronbach's alpha indicating a level of 0.75, signifying an acceptable degree of reliability.

Data analysis

In our analysis, we utilized IBM SPSS Statistics for Windows, Version 28.0 (Released 2021; IBM Corp., Armonk, New York, United States). Descriptive statistics were employed to compile and summarize the data. To delve deeper into the connection between knowledge, awareness of GDM, and demographic characteristics, we employed the chi-square test and logistic regression analysis. Significance levels were determined by a p-value less than 0.05; any finding with a p-value below this threshold was considered statistically significant.

Ethical consideration

This study received ethical approval from the Institutional Review Board of King Salman Armed Forces Hospital (approval number: KSAFH-REC-2023-513). Prior to their involvement in the trial, all participants provided oral informed consent. Questionnaires were collected from respondents while preserving their anonymity, emphasizing the importance of ethical considerations and participant confidentiality throughout the study.

## Results

Table 1 presents the demographic data, highlighting significant trends within the study participants. Notably, the age group of 30-39 years exhibited the highest frequency (N=206, 38.2%). Furthermore, the majority of participants had attained a university level of education (N=260, 48.2%). In terms of employment status, the highest frequency was observed among employed participants (N=170, 31.5%). Additionally, the study found that participants experiencing their first pregnancy (N=203, 37.7%) constituted a substantial portion of the sample.

**Table 1:** Sociodemographic information (N=539)

Variables	Classifications	N	%
Age	<20 years	10	1.9%
	20-29 years	145	26.9%
	30-39 years	206	38.2%
	40+ years	178	33.0%
Education Level	Elementary school	15	2.8%
	Middle school	45	8.3%
	High school	143	26.5%
	University	260	48.2%
	Higher education	69	12.8%
	No education	7	1.3%
Occupation	Employed	170	31.5%
	Homemaker	154	28.6%
	Student	71	13.2%
	Unemployed	144	26.7%
How many times have you been pregnant, including the current pregnancy?	1	203	37.7%
	2	105	19.5%
	3	56	10.4%
	4	72	13.4%
	More than 4	103	19.1%

In Table 2, it is evident that a substantial portion of the participants were aware of GDM (N= 410, 76.1%). Furthermore, the prevalent understanding among participants was that GDM signifies glucose intolerance first recognized during pregnancy (N= 382, 70.9%). The majority also identified obesity (N= 215, 39.9%) and a family history of diabetes (N= 200, 37.1%) as significant risk factors for GDM. Concerning complications, 34% of participants recognized preeclampsia as a major concern for mothers with GDM (N= 183), followed by pregnancy-induced hypertension (N= 147, 27.3%). Interestingly, most participants identified macrosomia as a significant complication for babies born to mothers with GDM (N= 247, 45.8%).

**Table 2:** Gestational diabetes mellitus (GDM) knowledge

Question	Response	N	%
Have you ever heard of GDM?	Yes	410	76.1%
	No	129	23.9%
Which of the following is a definition of GDM?	A condition in which a woman has high blood pressure during pregnancy	90	16.7%
	A type of cancer that affects pregnant women	26	4.8%
	Glucose intolerance with onset or first recognition during pregnancy	382	70.9%
	None of the above	41	7.6%
Which of the following are risk factors for developing GDM?	Advanced maternal age	67	12.4%
	Family history of diabetes	200	37.1%
	Obesity	215	39.9%
	Smoking	6	1.1%
	None of the above	51	9.5%
Which of the following are possible complications of GDM for the mother?	Cesarean delivery	112	20.8%
	Postpartum depression	29	5.4%
	Pre-eclampsia	183	34.0%
	Pregnancy-induced hypertension	147	27.3%
	None of the above	68	12.6%
Which of the following are possible complications of GDM for the baby?	Hypoglycemia	97	18.0%
	Macrosomia	247	45.8%
	Respiratory distress syndrome	32	5.9%
	Shoulder dystocia	82	15.2%
	None of the above	81	15.0%

Table 3 presents the attitudes and beliefs of participants regarding the management of GDM during pregnancy. The majority of participants emphasized the crucial importance of managing GDM during pregnancy (N= 493, 91.4%). When considering strategies for management, dietary modifications were widely perceived as the most effective tool (N= 320, 59.4%). Additionally, a significant portion of participants expressed high confidence in their ability to adhere to the recommended management strategies (N= 356, 66%). Notably, 221 (41%) participants recognized the collective significance of all the mentioned factors, underlining the multifaceted approach necessary for successfully managing GDM.

**Table 3:** Attitude toward gestational diabetes mellitus (GDM) management

Question	Response	N	%
How important do you think it is to manage GDM during pregnancy?	Extremely important	344	63.8%
	Somewhat important	39	7.2%
	Very important	149	27.6%
	Not at all important	7	1.3%
Which of the following management strategies have you heard of for GDM?	Dietary modifications	320	59.4%
	Insulin therapy	123	22.8%
	Physical activity	70	13.0%
	None of the above	26	4.8%
How confident are you in your ability to follow the recommended management strategies for GDM?	Extremely confident	138	25.6%
	Somewhat confident	150	27.8%
	Very confident	218	40.4%
	Not at all confident	33	6.1%
Which of the following factors do you think are most important for successfully managing GDM?	Access to healthcare services	73	13.5%
	Knowledge about GDM	128	23.7%
	Motivation to manage GDM	38	7.1%
	Support from family and friends	79	14.7%
	All of the above	221	41.0%

Table 4 illustrates the sources of information and perceived barriers among participants. Healthcare providers emerged as the most prevalent source of information (N= 184, 34.1%), underscoring the significance of professional guidance. The Internet and social media followed closely (N= 171, 31.7%), indicating the growing influence of online platforms in disseminating health-related knowledge. Regarding barriers, a lack of knowledge about GDM stood out as the most common obstacle (N= 264, 49%), highlighting the need for comprehensive awareness campaigns. Limited access to healthcare services was another substantial barrier experienced by participants (N= 142, 26.3%), emphasizing the importance of enhancing healthcare accessibility to support effective GDM management strategies.

**Table 4:** Sources of information about gestational diabetes mellitus (GDM) and barriers to effective GDM management

Question	Response	N	%
Have you received information about GDM from any of the following sources?	Family or friends	105	19.5%
	Healthcare provider (e.g., doctor, nurse)	184	34.1%
	Internet or social media	171	31.7%
	Other	79	14.7%
How helpful do you find the information you received about GDM from healthcare providers?	Extremely helpful	178	33.0%
	Somewhat helpful	160	29.7%
	Very helpful	167	31.0%
	Not at all helpful	34	6.3%
Which of the following do you think are barriers to effectively managing GDM?	Cultural beliefs and practices	117	21.7%
	Lack of knowledge about GDM	264	49.0%
	Lack of support from family and friends	16	3.0%
	Limited access to healthcare services	142	26.3%

Table 5 presents a noteworthy association between knowledge and attitude among participants concerning GDM. When asked if they had ever heard of GDM, a highly significant association was found among the participants' responses (p=<0.001). The majority demonstrated a profound understanding of GDM, with 341 (89.3%) patients recognizing it as glucose intolerance first recognized during pregnancy. Similarly, 171 (79.5%) identified obesity as a significant risk factor for GDM. Participants overwhelmingly acknowledged the paramount importance of managing GDM during pregnancy, with 307 (89.2%) emphasizing its extreme significance. In terms of management strategies, 291 (90.9%) participants recognized dietary modifications as crucial. Healthcare providers, including doctors and nurses, were the primary source of information for 175 (95.1%) participants. Additionally, 240 (90.9%) cited a lack of knowledge as the most prevalent barrier to effectively managing GDM. The findings underscore a high level of knowledge and positive attitudes among participants who were familiar with GDM (N= 410, 76.1%).

**Table 5:** Association between knowledge, attitude of the participants, and their responses to whether they had ever heard of gestational diabetes mellitus (GDM) ^* ^P<0.001 is statistically significant

Question	Response	Have you ever heard of GDM?			
		N	Yes	No	*P-value
		539	410 (76.1%)	129 (23.9%)	<0.001
Which of the following is a definition of GDM?	A condition in which a woman has high blood pressure during pregnancy	90	36 (40%)	54 (60%)	<0.001
	A type of cancer that affects pregnant women	26	9 (34.6%)	17 (65.4%)	
	Glucose intolerance with onset or first recognition during pregnancy	382	341 (89.3%)	41 (10.7%)	
	None of the above	41	24 (58.5%)	17 (41.5%)	
Which of the following are risk factors for developing GDM?	Advanced maternal age	67	30 (44.8%)	37 (55.2%)	<0.001
	Family history of diabetes	200	166 (83%)	34 (17%)	
	Obesity	215	171 (79.5%)	44 (20.5%)	
	Smoking	6	5 (83.3%)	1 (16.7%)	
	None of the above	51	38 (74.5%)	13 (25.5%)	
Which of the following are possible complications of GDM for the mother?	Cesarean delivery	112	101 (90.2%)	11 (9.8%)	<0.001
	Postpartum depression	29	16 (55.2%)	13 (44.8%)	
	Pre-eclampsia	183	131 (71.6%)	52 (28.4%)	
	Pregnancy-induced hypertension	147	103 (70.1%)	44 (29.9%)	
	None of the above	68	59 (86.8%)	9 (13.2%)	
Which of the following are possible complications of GDM for the baby?	Hypoglycemia	97	73 (75.3%)	24 (24.7%)	<0.001
	Macrosomia	247	211 (85.4%)	36 (14.6%)	
	Respiratory distress syndrome	32	26 (81.3%)	6 (18.7%)	
	Shoulder dystocia	82	30 (36.6%)	52 (63.4%)	
	None of the above	81	70 (86.4%)	11 (13.6%)	
How important do you think it is to manage GDM during pregnancy?	Extremely important	344	307 (89.2%)	37 (10.8%)	<0.001
	Somewhat important	39	14 (9.1%)	25 (35.9%)	
	Very important	149	89 (59.7%)	60 (40.3%)	
	Not at all important	7	0	7 (100%)	
Which of the following management strategies have you heard of for GDM?	Dietary modifications	320	291 (90.9%)	29 (9.1%)	<0.001
	Insulin therapy	123	92 (74.8%)	31 (25.2%)	
	Physical activity	70	14 (20%)	56 (80%)	
	None of the above	26	13 (50%)	13 (50%)	
How confident are you in your ability to follow the recommended management strategies for GDM?	Extremely confident	138	120 (87%)	18 (13%)	<0.001
	Somewhat confident	150	99 (66%)	51 (34%)	
	Very confident	218	181 (83%)	37 (17%)	
	Not at all confident	33	10 (30.3%)	23 (69.7%)	
Which of the following factors do you think are most important for successfully managing GDM?	Access to healthcare services	73	52 (71.2%)	21 (28.8%)	<0.001
	Knowledge about GDM	128	94 (73.4%)	34 (26.6%)	
	Motivation to manage GDM	38	22 (57.9%)	16 (42.1%)	
	Support from family and friends	79	32 (40.5%)	47 (59.5%)	
	All of the above	221	210 (95%)	11 (5%)	
Have you received information about GDM from any of the following sources?	Family or friends	105	82 (78.15)	23 (21.9%)	<0.001
	Healthcare provider (e.g., doctor, nurse)	184	175 (95.1%)	9 (4.9%)	
	Internet or social media	171	112 (65.5%)	59 (34.5%)	
	Other	79	41 (51.9%)	38 (48.1%)	
How helpful do you find the information you received about GDM from healthcare providers?	Extremely helpful	178	157 (88.2%)	21 (11.8%)	<0.001
	Somewhat helpful	160	99 (61.9%)	61 (38.1%)	
	Very helpful	167	133 (79.6%)	34 (20.4%)	
	Not at all helpful	34	21 (61.8%)	13 (38.2%)	
Which of the following do you think are barriers to effectively managing GDM?	Cultural beliefs and practices	117	84 (71.8%)	33 (28.2%)	<0.001
	Lack of knowledge about GDM	264	240 (90.9%)	24 (9.1%)	
	Lack of support from family and friends	16	16 (100%)	0	
	Limited access to healthcare services	142	70 (49.3%)	72 (50.7%)	

## Discussion

GDM has attracted increasing attention among healthcare providers as a result of the growing incidence of the resultant development of T2DM during pregnancy and after childbirth among GDM patients. Preventing the diabetes epidemic involves detecting it during pregnancy. Therefore, it's critical that expectant mothers understand the risks of GDM and how to avoid them. Growing evidence from research shows that GDM care can be improved and its complications can be minimized with the help of GDM education and awareness campaigns in Primary Health Centers and patients' enhanced commitment to their own well-being.

Our study included 539 participants, the majority of whom were in the age group of 30-39 years (N= 206, 38.2%). With regard to education level, the majority were in university (N= 260, 48.2%); therefore, a significant number of the respondents had basic knowledge and could read. The employed participants showed the highest frequency (N= 170, 31.5%) as well as the once-pregnant participants (N= 203, 37.7%).

In the present study, the majority of the participants had good knowledge about GDM (N= 410, 76.1%). According to Dhyani et al.'s study in Karnataka, India, 57.6% of women had an average understanding of GDM, whereas 21.8% had strong knowledge and 19% had low knowledge [17]. Only 35.2% of people, according to Mahalakshmi et al. [18], and only 17.5% of women in Shreeram et al.'s study, had adequate awareness of GDM [16]. In multiethnic cohort research by Carolan, Indian women scored highest across all areas of interest, while Vietnamese and Filipino women and Caucasian women performed badly on general knowledge about GDM [19].

Knowledge regarding the risk factors associated with GDM was also assessed because this determines an individual’s ability to prevent or manage the condition. Of the participants, 71.9% (N= 382) knew that glucose intolerance with onset or first recognition during pregnancy is the major definition of GDM. Moreover, the majority of the participants saw that obesity (N= 215, 39.9%) and a family history of diabetes (N= 200, 37.1%) represented the major risk factors of GDM. Of the participants, 34% (N= 183) saw that preeclampsia represented the major complication of GDM in mothers, followed by pregnancy-induced hypertension (N= 147, 27.3%).

With regard to future impacts on the child of a mother with GDM, the majority of the participants revealed that macrosomia represented the major complication of GDM for babies (N= 247, 45.8%). In agreement with us, a related study in Nigeria found that 35.8% of participants were knowledgeable about the definition, risk factors, diagnosis, management, and consequences of GDM [20]. In India, it was found that 35.2% and 21.5% of moms who attended antenatal clinics had an adequate understanding of GDM and associated risk factors, respectively [21].

The main source of information in the present study was healthcare providers (N= 184, 34.1%), followed by the Internet or social media (N= 171, 31.7%). In the study by Dhyani et al., doctors (37%) and family members (22%) were the primary sources of information regarding knowledge about GDM [17]. Printed and electronic media were deemed to be the most significant sources of knowledge by Thomas et al. and Mahalakshmi et al. [22,18]. Social contacts, educational and professional experiences, and family history of diabetes and GDM were additional sources of information. While this study addressed a critical issue impacting pregnant women in Tabuk City, certain limitations must be acknowledged. The primary limitation lies in the study's utilization of a cross-sectional design, which, by its nature, establishes associations between variables rather than causality. This design restricts the ability to infer cause-and-effect relationships between exposure and outcome variables, limiting the depth of the study's conclusions. Researchers and readers should interpret the findings with this limitation in mind, understanding the constraints inherent in the chosen research approach.

## Conclusions

The Saudi women involved in this study demonstrated a commendable understanding of GDM, encompassing its definition and associated risk factors. Their attitudes toward GDM management and lifestyle modifications aimed at reducing complications were notably positive. Participants provided responses that were not only detailed but also logically sound, especially concerning sources of information about GDM and barriers hindering effective management.

Moreover, a significant finding emerged from the study: a highly substantial correlation between knowledge, attitude, and awareness of GDM among the participants (p = <0.001). This underscores the vital interconnection between being informed, having a positive mindset, and prior awareness, emphasizing the need for comprehensive education and awareness programs regarding GDM within the community. These results provide valuable insights into the existing understanding and attitudes of Saudi women regarding GDM, paving the way for targeted interventions and education initiatives.

## Appendices

**Table 6:** All sections of the questionnaire GDM: gestational diabetes mellitus

Section A: Demographic Information	
Variables	Classifications
Age	<20 years
	20-29 years
	30-39 years
	40+ years
Education Level	Elementary school
	Middle school
	High school
	University
	Higher education
	No education
Occupation	Employed
	Homemaker
	Student
	Unemployed
How many times have you been pregnant, including the current pregnancy?	1
	2
	3
	4
	More than 4
Section B: Gestational Diabetes Mellitus (GDM) Knowledge	
Question	Response
Have you ever heard of GDM?	Yes
	No
Which of the following is a definition of GDM?	A condition in which a woman has high blood pressure during pregnancy
	A type of cancer that affects pregnant women
	Glucose intolerance with onset or first recognition during pregnancy
	None of the above
Which of the following are risk factors for developing GDM?	Advanced maternal age
	Family history of diabetes
	Obesity
	Smoking
	None of the above
Which of the following are possible complications of GDM for the mother?	Cesarean delivery
	Postpartum depression
	Pre-eclampsia
	Pregnancy-induced hypertension
	None of the above
Which of the following are possible complications of GDM for the baby?	Hypoglycemia
	Macrosomia
	Respiratory distress syndrome
	Shoulder dystocia
	None of the above
Section C: Attitudes Towards GDM Management	
Question	Response
How important do you think it is to manage GDM during pregnancy?	Extremely important
	Somewhat important
	Very important
	Not at all important
Which of the following management strategies have you heard of for GDM?	Dietary modifications
	Insulin therapy
	Physical activity
	None of the above
How confident are you in your ability to follow the recommended management strategies for GDM?	Extremely confident
	Somewhat confident
	Very confident
	Not at all confident
Which of the following factors do you think are most important for successfully managing GDM?	Access to healthcare services
	Knowledge about GDM
	Motivation to manage GDM
	Support from family and friends
	All of the above
Section D: Sources of Information about GDM and Barriers to Effective GDM Management	
Question	Response
Have you received information about GDM from any of the following sources?	Family or friends
	Healthcare provider (e.g., doctor, nurse)
	Internet or social media
	Other
How helpful do you find the information you received about GDM from healthcare providers?	Extremely helpful
	Somewhat helpful
	Very helpful
	Not at all helpful
Which of the following do you think are barriers to effectively managing GDM?	Cultural beliefs and practices
	Lack of knowledge about GDM
	Lack of support from family and friends
	Limited access to healthcare services
